# The feasibility study of transnasopharyngeal esophagus echocardiography in the ultrasonic diagnosis

**DOI:** 10.1186/s12947-019-0154-2

**Published:** 2019-03-01

**Authors:** Xiaofeng Wang, Fang Nie, Na Ye, Xuehui Liu, Shaoqing Yang

**Affiliations:** Lanzhou University Second Hospital, Linxia Road, Chengguan District, Lanzhou City, 730030 Gansu Province China

**Keywords:** Trans nose pharynx esophagus echocardiography (TNPEE), Trans oral and esophageal echocardiography (TOEE)

## Abstract

**Background:**

Professor Xinfang Wang first introduced the clinical application of trans nose pharynx esophagus echocardiography (TNPEE) in 2014. Subsequently, we developed the technology. In the present study we assess the feasibility of TNPEE in the ultrasonic diagnosis.

**Methods:**

Select patients suitable for TNPEE examination. After obtaining written consent of patients or their families, oral dacronin hydrochloride gel local anesthesia was given 10–15 min before examination. The nostrils were disinfected and then smeared with tetracaine hydrochloride gel, which acted as local anesthesia and lubrication. The probe was inserted gently through the nostrils and entered the esophagus through the nasal cavity and pharynx. TNPEE is similar to transoral esophagus echocardiography (TOEE) after the probe reaches the esophagus.

**Results:**

TNPEE was performed in 103 patients. Forty-five patients (43.7%) underwent the examination successfully, 46 patients (44.7%) failed because of objective reasons, 12 patients midway refused to accept the examination and cancelled the examination, accounting for 11.6%, 11 patients (12.1%) suffered from epistaxis. Of all the patients with epistaxis, 9 had taken anticoagulant drugs, accounting for 82% of the patients with epistaxis. The vital signs of all patients were stable and no serious complications occurred.

**Conclusion:**

Compared with TOEE, TNPEE can cause less nausea and vomiting reaction, and patients take longer time to undergo examination, which is conducive to more detailed examination. However, TNPEE has a high requirement for the probe, and its success rate is relatively low. It is easy to cause nasal bleeding in patients, so its wide clinical application is limited.

## Background

In 1976, Frazin first introduced M-mode transesophageal echocardiography(TEE) probe with single crystal in oral cavity. In 1982, Schluter et al. in Germany used transoral phased array esophageal probe for examination. This is a great progress in TEE research. After continuous improvement, it has developed from two-dimensional ultrasound to three-dimensional ultrasound. Combining with advanced technologies such as color Doppler, this technique has developed rapidly [1]. At present, TEE is becoming more and more mature, which plays an important role in clinical diagnosis and treatment, and its application is more and more extensive. But this technology has its advantages and disadvantages. From October 31 to November 2, 2014, the 12th National Academic Conference on Echocardiography of Chinese Academy of Ultrasound Medical Engineering was held in Haikou City. Professor Xinfang Wang introduced the clinical application of trans nose pharynx esophagus echocardiography (TNPEE) for the first time. Our hospital has carried out this examination since November 2014. One hundred and-three people were examined in our hospital. This paper summarizes the problems and experiences encountered in this examination, and discusses the clinical value of TNPEE.

## Methods

### Study subjects

The study was approved by the Clinical Ethics Committee at the Second Hospital of the LanZhou University in GanSu. All participating patients were informed of the sensitivity, accuracy and limitations of the TNPEE. Written informed consent was obtained prior to enrollment.

From December 2014 to December 2015, 208 patients were examined by TEE. Among them, 103 were examined by TNPEE. There were 28 males and 33 females, aged 44–58 years, with an average of (53.14 + 4.19) years. Among them, 28 patients (74%) with atrial fibrillation, 10 patients (26%) with patent foramen ovale, 5 patients with valvular disease, and 5 patients after prosthetic valve replacement. All patients underwent routine TTE and other related examinations before operation to exclude contraindications of esophageal echocardiography. No eating or drinking within 8 h before the examination, no smoking. The preparation for TEE examination in conscious patients includes inquiring about the history of gastrointestinal and esophageal diseases. In addition to emergencies, such as aortic dissection, TEE should not be performed until at least 4 h after fasting. Operating room should be equipped with oxygen supply, sputum aspiration, blood pressure monitoring and resuscitation equipment. A finger oximeter can be used to monitor oxygen saturation. The left lateral decubitus position should be adopted for conscious patients in TEE examination so as to remove secretions easily and reduce the risk of tracheal inhalation. All patients who met the requirements of the examination were interviewed and signed before the examination, and written consent was obtained. The patient was given a dacronin hydrochloride syrup 15 min before the examination. During the examination, the patient’s nasal cavity and probe were smeared with lidocaine syrup for surface anesthesia and lubrication. The probe scanning surface is parallel to the face and slowly enters the nasopharynx, pharynx and esophagus.

### Instruments and methods

**Fig. 1:**
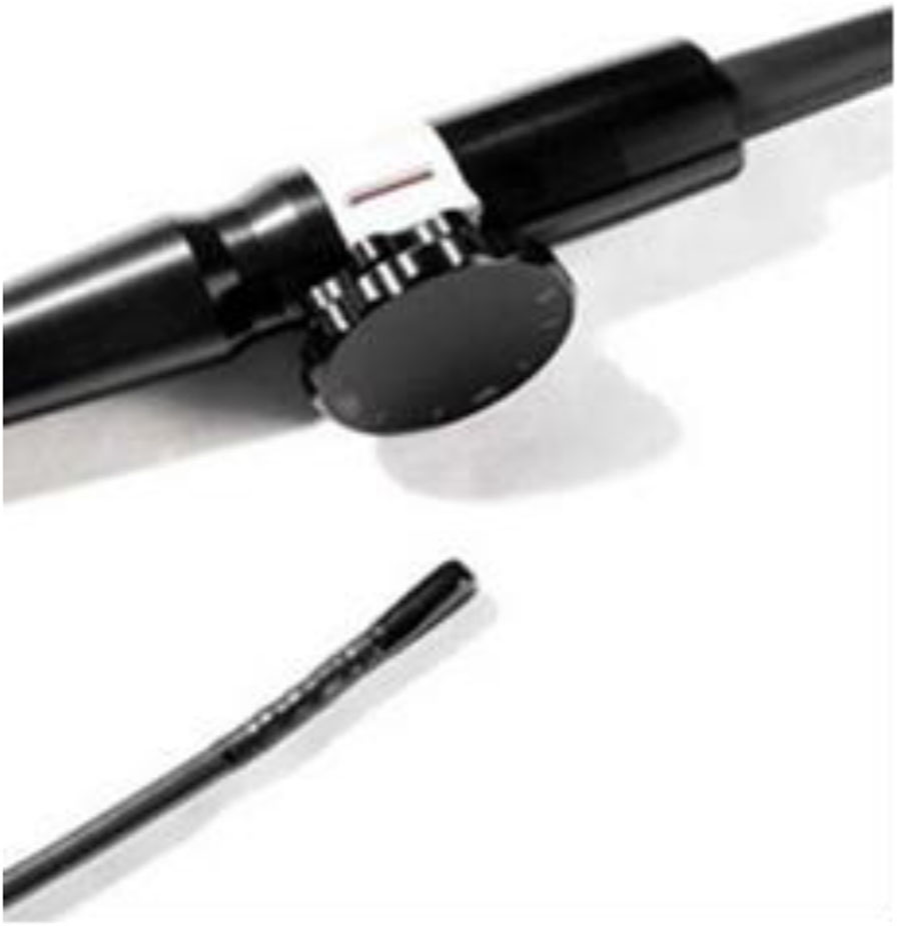
Philip S8-3 t probe

**Fig. 2:**
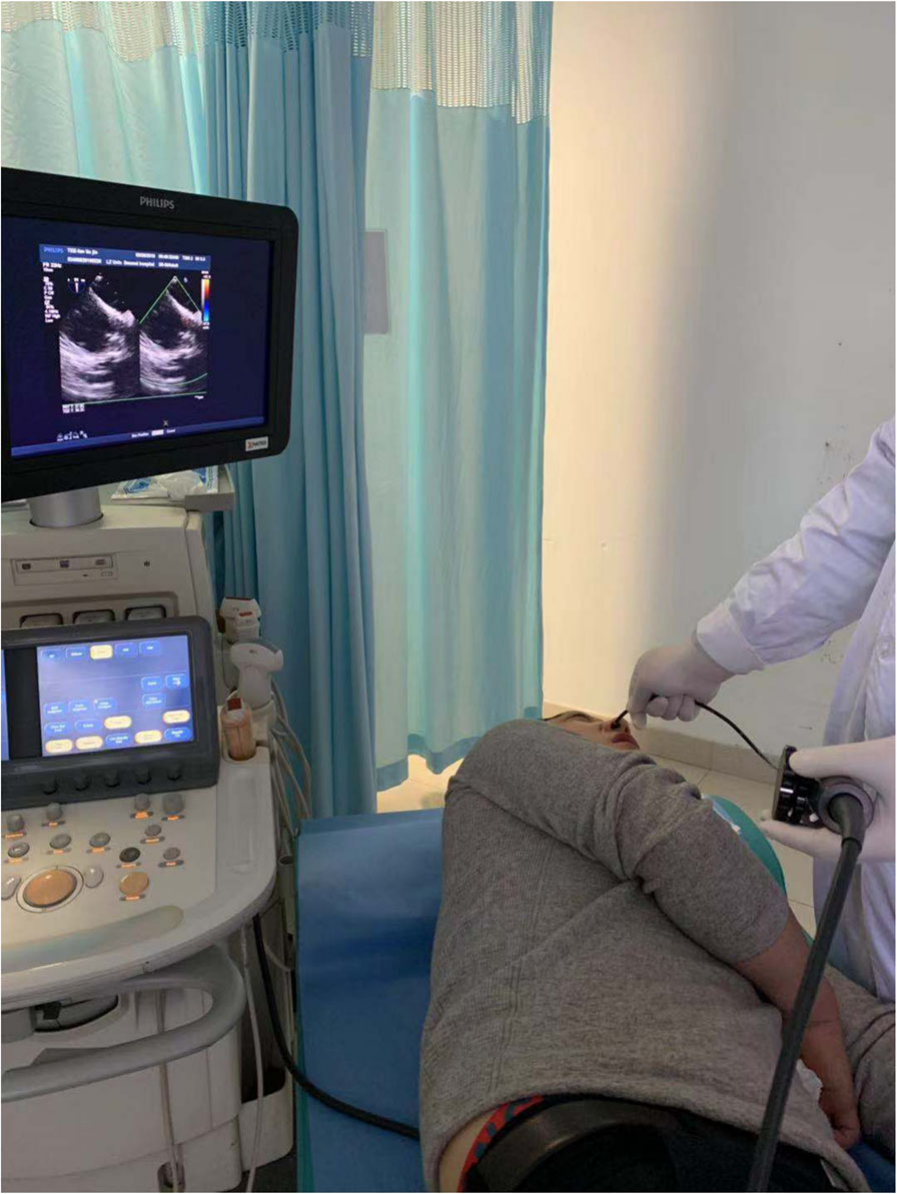
Transnasal echocardiography

Using Philip IE33 color Doppler ultrasound diagnostic instrument, the probe is Philip S8-3 t (Fig. 1). Examination process: (I) Pre-examination consultation: ① history of drug allergy; ② history of chronic rhinitis, nasal surgery, hypertrophy of turbinate, deviation of nasal septum and recent epistaxis, etc.; ③ Whether nosebleeds often occur in peacetime; ④ whether hemorrhages are easy to stop; ⑤ whether chronic diseases such as cirrhosis and uremia are suffered; ⑥ whether anticoagulants are taken for a long time. If the patient has the above situation, it may lead to the failure of TNPEE. (2) Half of lidocaine or one of dacronin hydrochloride were given orally 15 min before the start of the examination; (3) 10 min before the start of the examination, spray the normal saline into two nostrils with a nasal spray, 2–3 times on each side. Five minutes before the examination, vasoconstrictors were sprayed into both nasal cavities and breath tests were performed. The patients were asked to block the left nasal cavity and use the right nasal cavity to breathe, and then block the right nasal cavity and use the left nasal cavity to breathe. To compare which side of nasal airway is more smooth, the nasal cavity of the side is selected to enter the probe. Spraying both sides of the nasal cavity is to test which side of the nasal cavity is more suitable for inserting probe, and on the one hand can prevent nasal bleeding, on the other hand help to expand the nasal passage. (4) Inject anesthetics, 5 min before the beginning of the examination, and put 2 drops of furosemide into the selected nostrils, and 2% lidocaine hydrochloride was sprayed into the nasal cavity for local anesthesia. The lidocaine mucilage was slowly injected into the nasal cavity by 2–3 ml, The drug is topical anesthesia and the patient is conscious. (5) Before starting the examination, insert a cotton swab coated with 2% lidocaine hydrochloride into the nasal cavity to explore the inner diameter of the nasal cavity, stay in the nasal cavity for about 1 min to enhance local anesthesia and lubricate the nasal cavity, this will help the probe to insert through the nasopharynx. (6) During the examination, lidocaine was applied to the front of the esophageal probe, the probe was held in the right hand and gently inserted into the nasal cavity. The probe was facing the patient’s back. Generally, it can enter the lower or middle nasal meatus slowly. When it reaches the pharynx, the patient is instructed to swallow. When the probe enters the esophageal cavity, the other procedures are the same as those of the ordinary TOEE (Fig. 2). (7) After the examination, the probe was pulled out slowly. After 60 min, the patients could drink and eat. Because of the use of topical anesthesia, patients can drive.

## Results

One hundred and-three patients selected for TNPEE. Fifty-one patients (49.5%) successfully entered the esophagus through the nasal cavity, among them, 37 cases (35.9%) passed through the probe successfully through the single nostril, 14 cases (13.6%) successfully passed through the probe after replacing another nostril. Forty-five patients (43.7%) successfully completed the examination and achieved the purpose of the examination. Among them, 6 patients had epistaxis during the examination, and the examination was interrupted, accounting for 5.8%. After multi-angle adjustment, the probe could not pass through the left or right nasal cavity in 40 patients, accounting for 38.8%. All kinds of preparations have been completed. When the probe is ready to be placed, 12 cases (11.7%) feel very frightened when they see the probe and suddenly change their mind and refuse to enter the probe through the nasal cavity.

Among 103 patients, except 12 patients who refused to accept TNPEE examination midway, 91 patients received TNPEE, and cooperated well, accounting for 88%. Of the 91 patients examined, 45 completed the examination successfully, accounting for 49% of the patients examined; 46 patients failed because of objective reasons, accounting for 51% of the patients examined.

Forty-five patients who successfully completed the examination were followed up for 1 week after the examination, all patients had stable vital signs, no nasal and esophageal bleeding, no nasal obstruction and dysphagia. Among them, 30 cases had different degrees of nasal mucus or sputum bleeding, accounting for 67% of the patients who successfully completed the examination. The remaining 15 cases did not find nasal mucus or sputum bleeding, accounting for 33%.

Among all the patients examined, 11 cases (12%) suffered from nasal hemorrhage, of which 5 cases were terminated when the probe failed to pass through the nasal cavity; 6 cases were terminated during the examination, when the probe has entered into the esophagus through the nasal cavity. Among 11 patients with massive nasal hemorrhage, 9 patients with atrial fibrillation had a history of taking anticoagulants, accounting for 82% of patients with massive nasal hemorrhage, while the other 2 patients had no obvious objective inducement. Eleven patients with massive nasal hemorrhage were followed up, 10 patients were treated immediately by clinicians, the hemorrhage stopped on the same day, no repeated bleeding or other discomfort was found during the follow-up for 1 week. One patient had bleeding for 1 week, the clinician had nasal packing, compression and medication. After 1 week, the bleeding stopped. Follow-up showed that the patient had coagulation disorder (the patient did not know, the clinician neglected the relevant tests).

Among the 45 patients who successfully completed the examination, 7 had left atrial appendage thrombosis, 30 had patent foramen ovale with atrial septal shunt, 1 had aortic valve vegetation, 1 had perivalvular leak of mitral valve prosthesis, and 1 had thrombosis of mitral valve prosthesis, the others had no obvious abnormalities. Table 1.

**Table 1:** Comparison between transoral esophageal echocardiography (TOEE) and transnasopharyngeal esophageal echocardiography (TNPEE)

General information	TOEE	TNPEE
Number of patients	105 people	103 people
Age range	7~78 years	44~58 years
Male/ female	41/64	50/53
Complications	Mucosal bleeding (33% incidence); Recurrent pharyngitis (13% incidence); Other (5% incidence)	Mucosal bleeding(67% incidence); Recurrent pharyngitis (2% incidence); Other (1% incidence)
success rate	99%	49%
Positive rate	87%	89%

## Discussion

The traditional method of TEE is to insert the esophageal probe into the patient’s esophagus through the mouth and observe the internal structure and function of the heart from the back to the front. The frequency of esophageal probe is higher than that of transthoracic probe, which avoids the interference of chest wall, lung gas and obesity. The image clarity and resolution are better than that of transthoracic echocardiography (TTE). It improves the sensitivity and reliability of diagnosis of cardiovascular diseases and has become an important method for evaluating cardiac structure and function [2] . However, when it comes to esophageal echocardiography, people always think of a large esophageal probe passing through the throat, severe vomiting, painful expressions and saliva crossflow. Some patients refuse to be examined for fear of discomfort in the process of examination, which leads to the inability to make a definite diagnosis and take effective treatment measures [3] .

At present, conventional esophageal echocardiography often causes severe vomiting in patients due to its thick probe diameter and large stimulation by inserting it into the esophagus through the mouth. Some patients even asked to stop the examination or pull out the probe by themselves because they could not bear it. In order to alleviate the pain of patients, some hospitals ask anesthesiologists for general anesthesia before examination, which is costly and increases the financial burden of patients [4]. Due to the large space in the oral cavity, surrounded by mucosa and soft tissue, there is no hard bone tissue, so the probe with a thick diameter is easy to pass into the esophagus, but when the probe pass through the pharynx, it stimulates the tongue root, which is easy to cause nausea and vomiting. TNPEE is a new technique to relieve the discomfort during TNPEE. An esophageal probe with a diameter of about 5.2 mm was inserted into the esophagus through the nasopharynx, the probe entered the esophagus along the posterior wall of the pharynx behind the soft palate. Without touching the root of the tongue, the patients had mild nausea and vomiting reaction. And before the probe is inserted into the nasal cavity, local anesthesia of the nasal mucosa can make the patient feel less discomfort of the nasal cavity and can endure a longer examinations. In addition, patients can talk with doctors during the examination, so that the examination process can be completed in a relaxed and pleasant environment. However, the nostril is small, the distance between turbinate and nasal septum is small, cartilage and skeletal tissue are all present, the mobility and expansion are small, and it is difficult for adult probes to enter. At present, there is no special probe for TNPEE examination. The children’s esophageal probe S8-3 t produced by Philips is of small size, with the front width of the probe about 7.5 mm and the thickness about 5.5 mm. The transverse section of the probe is elliptical, which is smaller than the width and thickness of the adult probe. The diameter of the probe is very thin and very soft. [5] The probe was used for TNPEE examination in our department.

Suitable population for TNPEE examination: 1. Patients with hypersensitivity to pharyngeal stimulation, severe malignant and vomiting reactions, and unable to be examined by transoral esophageal echocardiography, if the overall condition of the patient is good, TNPEE can be tried. 2. TNPEE is an effective method for patients with oral dysfunction or other oral diseases caused by burns, chemicals, etc. who really need TEE examination to make a definite diagnosis. 3. For patients with high mental stress, TNPEE can be considered to prevent mandibular joint dislocation or tooth damage probe during examination; 4. For patients with dentures, TNPEE can be used to prevent the effect of the esophageal probe on dentures.

TNPEE contraindications: Patients with severe cardiopulmonary disease and poor physique are unable to cooperate with TNPEE examination; Liver cirrhosis with severe esophageal varices, esophageal neoplasms, esophageal diverticulum or tracheoesophageal fistula, esophageal stricture, severe esophagitis or esophageal ulcer, unexplained dysphagia; Patients with shock symptoms or unconsciousness; Patients with severe hypertension or unstable blood pressure; Patients with severe mental illness, unable to cooperate with TNPEE Examiners; Patients with obvious thoracic or abdominal aortic aneurysms; Stroke patients; Patients with severe hemorrhagic diseases, with hemoglobin below 50 g/L or PT above 1.5 s; Patients with severe thoracic or spinal deformities; Patients over 80 years old, etc.

Advantages of TNPEE Examination: 1) The probe is small and soft, and does not cause loss of esophageal mucosa, and does not cause serious complications such as esophageal perforation and tear of esophageal mucosa; 2) The probe enters the esophagus through the nasopharynx and along the posterior pharyngeal wall at the posterior inferior part of the soft palate. Normally, it does not touch the root of tongue, avoids the reflex sensitive area of pharynx and larynx, and stimulates the soft palate mucosa slightly. The nausea and vomiting reactions of the patients are obviously alleviated; 3) Patient whose probe passes through nasopharynx and enters esophagus smoothly can generally cooperate well with doctor’s examination, talk face to face with doctor, and tell doctor about discomfort at any time, so as to facilitate operator to understand patient’s immediate situation, reduce patient’s tension and avoid accidents; 4) During TNPEE examination, the patient’s symptoms such as malignancy and vomiting were alleviated, and the examination time was long, the examining doctor could have sufficient time to observe the cardiac lesions in detail.

Defects of TNPEE Examination: 1) At present, there is no special ultrasound probe for TNPEE. Philips S8-3 t probe is a special transesophageal probe for children. Although the probe is thin and soft, it is only suitable for some patients. For many patients, the probe can not pass through the nasal cavity. The success rate of the probe passing through the nasal cavity smoothly into the esophagus is relatively low. 2) Most of the patients requiring TNPEE are adults. The inner diameter of esophagus is relatively large. The S8-3 t probe is thin and soft. The contact between the probe and the esophageal wall is poor, which affects the image clarity.3) TNPEE is a new technique developed in recent years. There are few reports on it, lacking relevant information and experience, and its operation is blindness.4) TNPEE is easy to cause epistaxis in patients, causing panic among doctors and patients, the examination can not be carried out normally and has to be cancelled. 5) Compared with traditional TOEE, TNPEE has no obvious advantages in image quality, diagnostic rate and diagnostic accuracy. Its application value and prospect are uncertain.

The reasons for failure of TNPEE were summarized as follows: The main reason is that the patient’s nasal cavity is narrow, deformed or too sensitive, causing the probe to fail to pass through the nasal cavity. In addition, excessive bending of the probe in the nasal cavity is also an important reason for the failure of the probe insertion. Nasal hemorrhage is the main cause of the termination of the examination. After summarizing the experience of success and failure, we realized that in order to successfully complete the TNPEE, we need to pay attention to the following aspects: (1) Operators should be familiar with the routine procedures and techniques of TOEE, and be familiar with the anatomical structure of the nasopharynx, and be familiar with various transnasal operation skills and precautions, so as to ensure that the probe is inserted into the esophagus through the nasopharynx for examination. (2) When the probe passes through the nasal cavity, pharynx and esophagus, it should be prevented from bending the probe excessively, avoid the discomfort caused by the angle of the probe, and even cause the tear or perforation of the esophageal wall [6]. (3) The patient’s nasal cavity and probe surface are coated with a small amount of lidocaine glue, which can play both a local surface anesthetic effect and a lubrication effect to protect the mucous membranes. (4) Some patients with hypertrophy of turbinate can insert probe through contralateral nasal cavity. If both turbinates are hypertrophic, it should be changed to traditional TOEE.

TNPEE is a new technique developed by referring to transnasogastric endoscopy technology. Hospitals that have reported the technology in China are few, and there is no relevant information and experience for reference. The biggest advantage of this technique is that it has less nausea and vomiting reaction and can tolerate longer examination. However, because there is no special TNPEE probe, the success rate of using Philips S8-3 t probe through the nasal cavity is low. Of the 103 patients selected in our hospital, only 51 patients passed through the nasal cavity smoothly, accounting for 49.5%, and the success rate is less than half. Of the 45 patients who successfully completed the examination, 30 had different degrees of nasal mucosal bleeding or sputum bleeding, accounting for 67% of the patients who successfully completed the examination, indicating that most people will have different degrees of nasal mucosal bleeding. 11 patients suffered from nasal hemorrhage, accounting for 12% of the patients examined. This indicates that TNPEE has a high risk of causing nasal hemorrhage.

## Conclusions

TNPEE is a method used for special population examination. It can not be used routinely in clinical practice. Related problems have been studied. To sum up, We believe that the advantages of this technique are not outstanding, the failure rate and the risk of epistaxis are high. Due to the lack of special probe for TNPEE and limited clinical application value, it is difficult to popularize it widely. With the progress of science and technology, if a more thin and soft TNPEE special probe is produced, the technology may be widely used.

## Abbreviations

TEE: Transoral esophageal echocardiography
TNPEE: Transnasopharyngeal esophageal echocardiography
TOEE: Transoral esophageal echocardiography
TTE: Transthoracic echocardiography
